# Risk of Herpes Zoster in Patients with Adhesive Capsulitis of the Shoulder

**DOI:** 10.3390/ijerph17103592

**Published:** 2020-05-20

**Authors:** Chao-Yu Hsu, Der-Shin Ke, Cheng-Li Lin, Chia-Hung Kao

**Affiliations:** 1 Department of Medical Education, Ditmanson Medical Foundation, Chia-Yi Christian Hospital, Chia-Yi 60002, Taiwan; hsuchaoyu66@yahoo.com (C.-Y.H.); matthew611@gmail.com (D.-S.K.); 2 Department of Family Medicine, Ditmanson Medical Foundation, Chia-Yi Christian Hospital, Chia-Yi 60002, Taiwan; 3 Department of Optometry, Central Taiwan University of Science and Technology, Taichung 40601, Taiwan; 4 Center for General Education, National Taichung University of Science and Technology, Taichung 40401, Taiwan; 5 The General Education Center, Chaoyang University of Technology, Taichung 41349, Taiwan; 6 Department of General Education, National Chin-Yi University of Technology, Taichung 41170, Taiwan; 7 Center for General Education, National Chi Nan University, Puli 54561, Taiwan; 8 Rural Generalist Program Japan, GENEPRO, Asahi Shi 289-2505, Japan; 9 Management Office for Health Data, China Medical University Hospital, Taichung 40447, Taiwan; orangechengli@gmail.com; 10 College of Medicine, China Medical University, Taichung 40447, Taiwan; 11 Graduate Institute of Biomedical Sciences and School of Medicine, College of Medicine, China Medical University, Taichung 40447, Taiwan; 12 Department of Nuclear Medicine and PET Center, China Medical University Hospital, Taichung 40447, Taiwan; 13 Department of Bioinformatics and Medical Engineering, Asia University, Taichung 41354, Taiwan; 14 Center of Augmented Intelligence in Healthcare, China Medical University Hospital, Taichung 40447, Taiwan

**Keywords:** herpes zoster, adhesive capsulitis of shoulder, diabetes, obesity, depression

## Abstract

*Background*: Physical diseases, such as infection, and emotional distress are associated with herpes zoster (HZ). However, the relationship between adhesive capsulitis of the shoulder (ACoS) and HZ remains unknown. *Objective*: This study investigated the risk of HZ development in patients with ACoS. *Methods*: We analyzed the Longitudinal Health Insurance Database, a subset of 1 million beneficiaries from the National Health Insurance Research Database. Patients newly diagnosed with ACoS during the 2000–2012 period were the case group. Each patient with ACoS was matched to a control, according to age and index year, through frequency matching. HZ was the primary event in this study. *Results*: A total of 60,478 patients were included and each group contained 30,239 patients. The risk of HZ infection in the case cohort was 1.28 times that of the control cohort. ACoS increased the risk of HZ infection in each age group, particularly among patients aged younger than 50 [adjusted hazard ratio (aHR) = 1.52, 95% confidence interval (CI): 1.31–1.75]. Relative to the control group, the hazard ratio of HZ for male patients (aHR = 1.40, 95% CI: 1.26–1.55) in the case group was higher than that for female patients (aHR = 1.22, 95% CI: 1.13–1.32). *Conclusion*: Patients with ACoS have a higher risk of HZ development, particularly among those aged younger than 50 years. The effect of ACoS on HZ development among young adults requires attention.

## 1. Introduction

Adhesive capsulitis of the shoulder (ACoS), also called frozen shoulder, is a painful condition that occurs when the connective tissue surrounding the glenohumeral joint of the shoulder becomes thick, stiff, and inflamed. The term “frozen shoulder” was first coined by Earnest Codman in 1934 to describe the gradual motion loss in the affected shoulder [1].

ACoS is categorized into primary (idiopathic) and secondary. Secondary ACoS is associated with fracture, dislocation, osteoarthritis or tendinopathy [2]. The prevalence of ACoS in the general population is between 2% and 5% [2]. It occurs more commonly among middle-aged people [1], particularly in women [2]. An effective treatment for ACoS has yet to be established; at present, conservative treatment (nonsteroidal anti-inflammatory medication, corticosteroid injection, physiotherapy, and hydrodilatation) and surgery (manipulation under anesthesia and arthroscopic capsule release) are both used [2,3].

Herpes zoster (HZ), a reactivation of the varicella-zoster virus, is characterized by painful vesicular rashes with a dermatomal distribution. HZ can affect people of all ages, with adults aged older than 50 years having an increased risk. Because of immune attenuation, this risk increases with age [4]. Wolfson et al. analyzed the Market Scan Commercial and Medicare databases and reported that HZ incidence in adults increased between 1991 and 2016. Among people aged between 18 and 65 years, the annual rates of change were 1.2% to 6.2%. However, for adults aged ≥65 years, the annual rates of change were 12.4% in 1991–1995, 3.6% in 1996–2006 and 1.7% in 2007–2016 [5]. Postherpetic neuralgia is a painful and disabling condition, and may occur for months to years after recovery from acute HZ.

Physical diseases [6,7,8], and emotional distress [9] are correlated with HZ. ACoS and ACoS-related pain cause stress in patients and may induce HZ development. Therefore, this study investigated the risk of HZ development in patients with ACoS.

## 2. Materials and Methods

### 2.1. Data Source

The data were acquired from the National Health Insurance Research Database (NHIRD), which was established on 1 March 1995, and covers almost the entire Taiwanese population. We analyzed the Longitudinal Health Insurance Database (LHID), a subset of the NHIRD’s 1 million beneficiaries. The LHID contains medical records such as outpatient visits, inpatient visits, medication, operation treatment, and other medical care data of all beneficiaries from 1996 to 2013. The disease codes conform to the International Classification of Diseases, Ninth Revision, Clinical Modification (ICD-9-CM).

### 2.2. Study Population

Patients newly diagnosed with ACoS (ICD-9-CM 726.0) during the 2000–2012 period comprised the case group. The control group included subjects without ACoS. We excluded patients with previous HZ and those aged younger than 20 years. One ACoS patient was matched to one control, according to age and index year (the diagnosis date of ACoS for case group; randomly assigned for control group), through frequency matching. All patients were followed up from the index date until HZ diagnosis, death, loss, or 31 December 2013, whichever came first.

### 2.3. Main Outcome and Comorbidities

HZ (ICD-9-CM 053) was the primary event of this study. The potential confounders were diabetes (ICD-9-CM 250), coronary artery disease (CAD; ICD-9-CM 410 to 414), depression (ICD-9-CM: 296.2, 296.3, 300.4, 311), chronic kidney disease (CKD; ICD-9-CM 585, 586), obesity (ICD-9-CM 278), and cancer (ICD-9-CM 140 to 208).

### 2.4. Statistical Analysis

Differences between the demographic characteristics and comorbidity distributions between the case and control groups were examined using the chi-square test and Mann–Whitney U-test. The value of median was presented as ± interquartile range (IQR). The incidence of HZ was calculated with the unit of 1000 person-years. A Cox proportional hazard regression model was used to estimate the crude hazard ratios of HZ for each factor. The hazard ratios were then adjusted by including age, sex, diabetes, CAD, depression, CKD, and cancer in the multivariable model. We used Kaplan–Meier methods to determine the cumulative incidences of HZ in the case and control groups and compared the results using a log-rank test. Results with *p* < 0.05 were considered statistically significant.

## 3. Results

A total of 60,478 patients were included, and each group contained 30,239 patients. The mean follow-up times for the case and control groups were 6.80 (± 3.74) years and 6.76 (± 3.77) years, respectively. The characteristics of the case and control groups are listed in the Table 1. The distributions of age and sex were similar in the two cohorts. The median age of the case group (56.3 years) was slightly higher than that of the comparison group (55.6 years). Compared with the control group, the case group included more patients with diabetes, CAD, depression, obesity, and cancer.

As listed in Table 2, the risk of HZ in the case cohort was 1.28 times (95% confidence interval (CI): 1.20–1.36) that of the control cohort. Compared with adults aged younger than 49 years, those aged 50 or older were more likely to develop HZ. The adjusted hazard ratio (aHR) of HZ for women relative to men was 1.09 (95% CI: 1.02–1.16). Patients with diabetes, CAD, depression, and CKD had a higher risk of developing HZ compared with those without these comorbidities.

**Figure 1:**
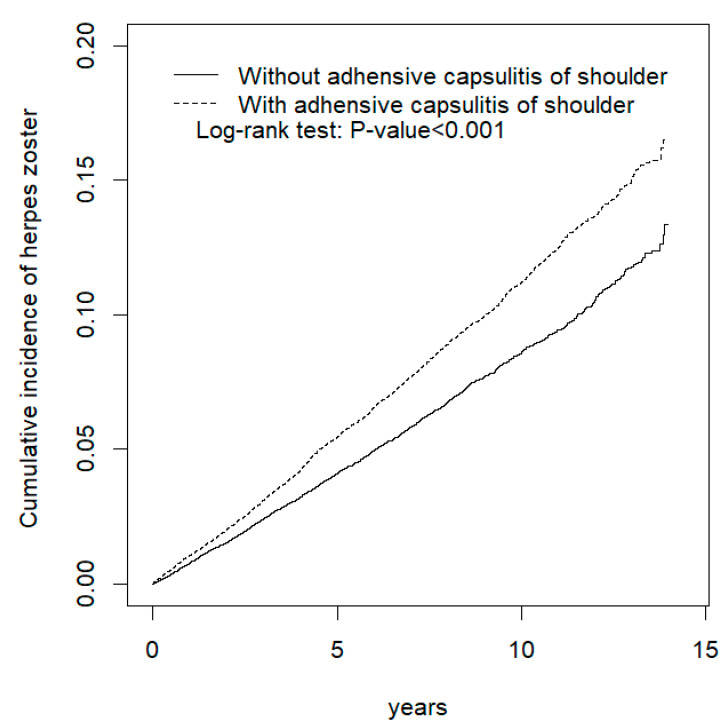
Cummulative incidence comparison of herpes zoster for patients with (dashed line) or without (solid line) adhesive capsulitis of shoulder. The unit of y axis (cumulative incidence) is %.

**Figure 2:**
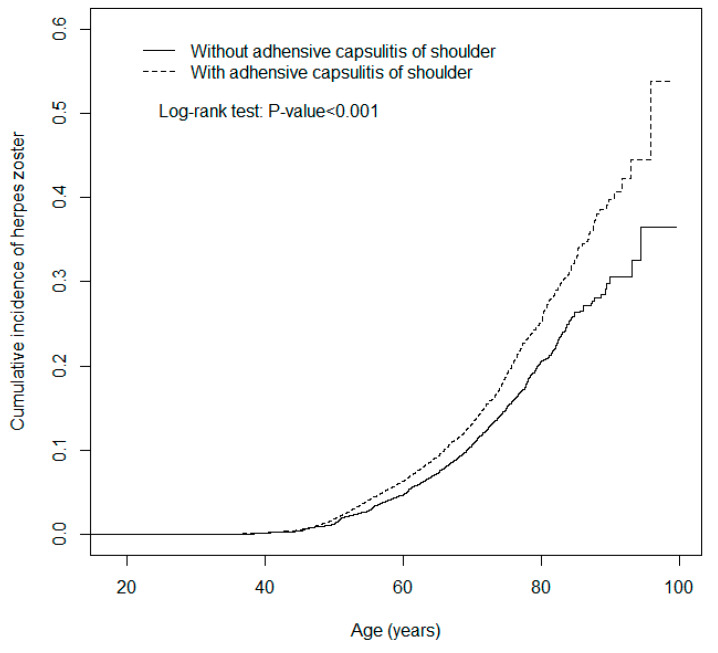
Cumulative incidence of herpes zoster for patients with (dashed line) or without (solid line) adhesive capsulitis of the shoulder versus index age. The unit of y axis (cumulative incidence) is %.

Table 3 lists the results of the stratification analysis. ACoS increased the risk of HZ infection in each age group, particularly among adults aged younger than 50 years (aHR = 1.52, 95% CI: 1.31–1.75). The hazard ratios of HZ for males (aHR = 1.40, 95% CI: 1.26–1.55) in the case group relative to control group was higher than that for females (aHR = 1.22, 95% CI: 1.13–1.32). Both with and without comorbidities, HZ development was significant and positive among the patients with ACoS. As illustrated in Figure 1, the cumulative incidence of HZ for patients with ACoS was significantly higher than that of controls (log-rank test: *p* value < 0.001). Even the risk for HZ between ACoS and non-ACoS groups was significantly higher through the ascending of HZ index age in Figure 2 (log-rank test, *p* < 0.001).

## 4. Discussion

This is the first population-based study to identify the association between ACoS and HZ infection. We noted that patients with ACoS were 1.28 times more likely than the controls to develop HZ.

The association between ACoS and diabetes has been thoroughly identified. The most common association with primary (idiopathic) ACoS is diabetes [2]. The prevalence of ACoS in the general population has been estimated to be between 2% and 4%, and it is reported to be significantly higher in patients with diabetes, with estimates ranging from 10% to 22% [10,11]. The rates of ACoS in patients with diabetes 1–5 and 5–10 years after diagnosis were 32.3% and 33.8%, respectively [12]. Huang et al. reported a hazard ratio of ACoS development in patients with diabetes compared with those without diabetes was 1.321, after adjusting for age, sex, and dyslipidemia [13]. In our study population, the proportion of patients with diabetes was significantly higher in the ACoS cohort than in the control cohort after matching by age and index year. However, it remains unclear whether we should screen patients with ACoS for diabetes.

Safran et al. reported that only 8% of patients with ACoS were suspected of having diabetes. They suggested that routine diabetes screening is not necessary, but only 50 patients were enrolled in their study [10]. A meta-analysis revealed that patients with diabetes are five times more likely to develop ACoS than those without diabetes. In addition, the prevalence of diabetes among the patients with ACoS was determined to reach 30%. Therefore, the authors suggested screening for diabetes should be considered for patients with ACoS [14]. Rai et al. reported rates of 15.5% and 27.4% for prediabetes and diabetes, respectively, among the patients with ACoS. The authors recommended diabetic screening for patients with ACoS, comprising at least fasting and prandial blood sugar tests [11]. The results of the present study agree with findings that patients with ACoS should receive blood glucose tests for diabetes as early as possible. Finding and treating diabetes earlier can prevent diabetes-associated side effects.

Several studies have attempted to identify the relationship between obesity, depression, and ACoS. Lin et al. noted that obesity was significantly higher in patients with ACoS than in their controls (23.6% vs. 18.4%) [15]. Similar findings were reported by Kingston et al., who revealed a 27% prevalence of obesity among patients with ACoS. Furthermore, they found that obesity was 1.26 times more common among patients with ACoS than among controls. Kingston et al. concluded that obesity is significantly associated with ACoS and should be considered as a modifiable risk factor [16]. In the present study, the prevalence of obesity was significantly higher in the case group than in the control group (Table 1). These results confirm that weight reduction would be beneficial for patients with ACoS.

Ding et al. noted a higher Hospital Anxiety and Depression Scale among patients with ACoS relative to their controls. They reported a 28.2% prevalence of depression among patients with ACoS and claimed that depression coexists with ACoS [17]. However, contradictory results were reported by Toprak and Erden. They found no significant difference between the Beck Depression Inventory scores of patients with ACoS and controls [18]. On the basis of the Hamilton anxiety and depression questionnaire, Ebrahimzadeh et al. observed a 77% prevalence of depression among patients with ACoS. However, they also reported no definitive relationship between depression and range of motion of the shoulder among the patients with ACoS. They suggested that depression among patients with ACoS was due to the experience of increased limb pain and disability [19]. The results of the present study support this finding; we noted more patients with depression in the case group than in the control group after matching by age and index year (Table 1). Clinicians should pay attention to the exacerbation of HZ due to depression caused by ACoS-related pain.

As we know, the risk of HZ increases with age due to immune attenuation [4]. Our findings are consistent with the results (Table 2). Relative to patients aged younger than 49 years, the aHR of HZ infection for those aged 50–64 years was 1.74 and the aHR for those aged 65 years and older was 2.22. However, among patients with ACoS, the aHR of HZ infection was significantly higher for patients aged ≤49 years (Table 3). Therefore, HZ development appears to be more affected by ACoS than by age. The effect of ACoS among young adults should not be ignored.

This population-based study was conducted using NHIRD records. The NHIRD covers a highly representative sample of the Taiwanese population because of its large sample size, allowing for highly generalizable results. However, this study had several limitations. First, some patients with ACoS may receive alternative therapies such as acupuncture or chiropractic. Under-diagnosis is also possible, because the NHIRD does not cover noninsured treatments, and self-payment for treatment is not recorded in the NHIRD. Second, because disease severity is not included in the NHIRD, the burden related to the disease severity could not be considered. Third, lifestyle-related factors, such as exercise or diet, are not captured in the NHIRD, despite potentially influencing immunity. Finally, specialists and general practitioners may have used different diagnostic codes. However, the National Health Insurance is administered by the Taiwanese government, and all insurance claims are sent to the National Health Insurance Administration and reviewed by insurance experts. Because of the strict system of audit and punishment, therefore, the diagnostic codes are reliable.

There are unknown mediators which may affect the association between ACoS and HZ. However, unknown mediators may only have weak effect. We only discussed the most important factors in the text. Despite these limitations, the study results accurately describe the target population. The results confirm that patients with ACoS have a higher risk of HZ development relative to those without ACoS.

## 5. Conclusions

Patients with ACoS have a higher risk of HZ development, particularly among those aged younger than 50 years. The effect of ACoS on HZ development among young adults requires attention.

## Figures and Tables

**Table 1:** Demographic characteristics and comorbidities in cohorts with and without adhesive capsulitis of the shoulder.

Variable	Adhesive Capsulitis of the Shoulder		*p*-Value
	No	Yes	
	N = 30,239	N = 30,239	
Age, year			0.99
≤49	8342 (27.6)	8342 (27.6)	
50–64	13,352 (44.2)	13,352 (44.2)	
65+	8545 (28.3)	8545 (28.3)	
Median (IQR) ^†^	55.6 (47.9–66.3)	56.3 (49.2–66.6)	<0.001
Sex			0.99
Female	18,433 (61.0)	18,433 (61.0)	
Male	11,806 (39.0)	11,806 (39.0)	
Comorbidity			
Diabetes	2553 (8.44)	4021 (13.3)	<0.001
CAD	5385 (17.8)	7570 (25.0)	<0.001
Depression	1591 (5.26)	2429 (8.03)	<0.001
Chronic kidney disease	647 (2.14)	690 (2.28)	0.23
Obesity	431 (1.43)	608 (2.01)	<0.001
Cancer	961 (3.18)	1062 (3.51)	0.02

Chi-Square Test; ^†^: Mann–Whitney U-test; CAD denotes coronary artery disease; IQR, interquartile range.

**Table 2:** The incidence and risk factors for herpes zoster.

Variable	Event	PY	Rate ^#^	Crude HR (95% CI)	Adjusted HR ^&^ (95% CI)
Adhesive capsulitis of the shoulder					
No	1817	204,507	8.88	1.00	1.00
Yes	2421	205,670	11.8	1.33 (1.25, 1.41) ***	1.28 (1.20, 1.36) ***
Age, year					
≤49	763	125,368	6.09	1.00	1.00
50–64	1956	179,862	10.9	1.81 (1.66, 1.97) ***	1.74 (1.60, 1.90) ***
65+	1519	104,976	14.5	2.43 (2.22, 2.65) ***	2.22 (2.03, 2.44) ***
Sex					
Female	2718	255,139	10.7	1.08 (1.02, 1.15) *	1.09 (1.02, 1.16) *
Male	1520	155,068	9.80	1.00	1.00
Comorbidity					
Diabetes					
No	3688	370,547	9.95	1.00	1.00
Yes	550	39,659	13.9	1.42 (1.29, 1.55) ***	1.11 (1.01, 1.21) *
CAD					
No	3069	327,661	9.37	1.00	1.00
Yes	1169	82,545	14.2	1.53 (1.43, 1.63) ***	1.16 (1.07, 1.24) ***
Depression					
No	3923	386,997	10.1	1.00	1.00
Yes	315	23,209	13.6	1.37 (1.22, 1.53) ***	1.20 (1.07, 1.34) **
Chronic kidney disease					
No	4126	403,717	10.2	1.00	1.00
Yes	112	6490	17.3	1.74 (1.44, 2.10) ***	1.35 (1.12, 1.63) **
Obesity					
No	4185	404,328	10.4	1.00	1.00
Yes	53	5878	9.02	0.89 (0.68, 1.17)	
Cancer					
No	4103	399,593	10.3	1.00	1.00
Yes	135	10,613	12.7	1.27 (1.07, 1.50) **	1.09 (0.92, 1.29)

Rate ^#^, incidence rate, per 1000 person-years; Crude HR, relative hazard ratio; Adjusted HR^&^: multivariable analysis including age, sex, and comorbidities of diabetes, CAD, depression, chronic kidney disease, and cancer; * *p* < 0.05, ** *p* < 0.01, *** *p* < 0.001.

**Table 3:** Incidence of herpes zoster by age, sex and comorbidity and Cox model measured hazards ratio for patients with adhesive capsulitis of the shoulder compared those without adhesive capsulitis of the shoulder.

	Adhesive Capsulitis of the Shoulder							
	No			Yes				
Variables	Event	PY	Rate ^#^	Event	PY	Rate ^#^	Crude HR (95% CI)	Adjusted HR ^&^ (95% CI)
Age, years								
≤49	300	62,903	4.77	463	62,465	7.41	1.56 (1.35, 1.80) ***	1.52 (1.31, 1.75) ***
50–64	855	89,760	9.53	1101	90,101	12.2	1.28 (1.17, 1.40) ***	1.26 (1.15, 1.38) ***
65+	662	51,843	12.8	857	53,133	16.1	1.26 (1.14, 1.40) ***	1.22 (1.10, 1.35) ***
Sex								
Female	1196	127,118	9.41	1522	128,021	11.9	1.26 (1.17, 1.36) ***	1.22 (1.13, 1.32) ***
Male	621	77,389	8.02	899	77,679	11.6	1.44 (1.30, 1.60) ***	1.40 (1.26, 1.55) ***
Comorbidity ^§^								
No	1165	151,759	7.68	1350	130,055	10.4	1.35 (1.25, 1.46) ***	1.36 (1.26, 1.47) ***
Yes	652	52,747	12.4	1071	75,644	14.2	1.14 (1.04, 1.26) **	1.16 (1.06, 1.28) **

Rate ^#^, incidence rate, per 1000 person-years; Crude HR, relative hazard ratio; Adjusted HR^&^: multivariable analysis including age, sex, and comorbidities of diabetes, CAD, depression, and chronic kidney disease; ^§^ Individuals with any comorbidity of diabetes, CAD, depression, and chronic kidney disease, obesity, and cancer were classified into the comorbidity group. ** *p* < 0.01, *** *p* < 0.001.

## References

[1] Le H.V., Lee S.J., Nazarian A., Rodriguez E.K. (2017). Adhesive capsulitis of the shoulder: Review of pathophysiology and current clinical treatments. Shoulder Elb..

[2] Cho C.H., Bae K.C., Kim D.H. (2019). Treatment strategy for frozen shoulder. Clin. Orthop. Surg..

[3] Ramirez J. (2019). Adhesive Capsulitis: Diagnosis and management. Am. Fam. Physician.

[4] Feli K.H., Ediale C.E., McMichael A.J. (2019). Update in herpes zoster prevention and the role of dermatologists. J. Drugs Dermatol..

[5] Wolfson L.J., Daniels V.J., Altland A., Black W., Huang W., Ou W. (2020). The impact of varicella vaccination on the incidence of varicella and herpes zoster in the United States: Updated evidence from observational databases, 1991–2016. Clin. Infect. Dis..

[6] Hsu C.Y., Lin C.L., Kao C.H. (2015). Balanitis is a risk factor for herpes zoster. Eur. J. Clin. Microbiol. Infect. Dis..

[7] Hsu C.Y., Wang Y.C., Kao C.H. (2015). Dyshidrosis is a risk factor for herpes zoster. J. Eur. Acad. Dermatol. Venereol..

[8] Hsu C.Y., Lin C.L., Kao C.H. (2020). Association between chronic interstitial cystitis and herpes zoster. Int. J. Environ. Res. Public Health.

[9] Liao C.H., Chang C.S., Muo C.H., Kao C.H. (2015). High prevalence of herpes zoster in patients with depression. J. Clin. Psychiatry.

[10] Safran O., El-Haj M., Leibowitz G., Beyth S., Furman Z., Milgrom C., Kandel L. (2017). Should patients with frozen shoulder be screened for diabetes mellitus?. Orthop. J. Sports Med..

[11] Rai S.K., Kashid M., Chakrabarty B., Upreti V., Shaki O. (2019). Is it necessary to screen patient with adhesive capsulitis of shoulder for diabetes mellitus?. J. Fam. Med. Prim. Care.

[12] Alhashimi R.A.H. (2018). Analytical observational study of frozen shoulder among patients with diabetes mellitus. Joints.

[13] Huang Y.P., Fann C.Y., Chiu Y.H., Yen M.F., Chen L.S., Chen H.H., Pan S.L. (2013). Association of diabetes mellitus with the risk of developing adhesive capsulitis of the shoulder: A longitudinal population-based followup study. Arthritis Care Res. (Hoboken).

[14] Zreik N.H., Malik R.A., Charalambous C.P. (2016). Adhesive capsulitis of the shoulder and diabetes: A meta-analysis of prevalence. Muscles Ligaments Tendons J..

[15] Li W., Lu N., Xu H., Wang H., Huang J. (2015). Case control study of risk factors for frozen shoulder in China. Int. J. Rheum. Dis..

[16] Kingston K., Curry E.J., Galvin J.W., Li X. (2018). Shoulder adhesive capsulitis: Epidemiology and predictors of surgery. J. Shoulder Elb. Surg..

[17] Ding H., Tang Y., Xue Y., Yang Z., Li Z., He D., Zhao Y., Zong Y. (2014). A report on the prevalence of depression and anxiety in patients with frozen shoulder and their relations to disease status. Psychol. Health Med..

[18] Toprak M., Erden M. (2019). Sleep quality, pain, anxiety, depression and quality of life in patients with frozen shoulder1. J. Back Musculoskelet. Rehabil..

[19] Ebrahimzadeh M.H., Moradi A., Bidgoli H.F., Zarei B. (2019). The relationship between depression or anxiety symptoms and objective and subjective symptoms of patients with frozen shoulder. Int. J. Prev. Med..

